# A systematic review of literature on Insulin‐like growth factor‐2‐mediated hypoglycaemia in non‐islet cell tumours

**DOI:** 10.1002/edm2.471

**Published:** 2024-02-27

**Authors:** Fateen Ata, Hassan Choudry, Adeel Ahmad Khan, Anum, Ibrahim Khamees, Anas Al‐sadi, Abdelaziz Mohamed, Lujain Malkawi, Esra'a Aljaloudi

**Affiliations:** ^1^ Department of Endocrinology and Metabolism, Hamad General Hospital Hamad Medical Corporation Doha Qatar; ^2^ Internal Medicine University Hospital of Coventry and Warwickshire (UHCW) Coventry UK; ^3^ Department of Internal Medicine Punjab Medical College/Faisalabad Medical University Faisalabad Pakistan; ^4^ Department of Internal Medicine University of Missouri‐Kansas City Kansas City Missouri USA; ^5^ Department of Internal Medicine, Hamad General Hospital Hamad Medical Corporation Doha Qatar; ^6^ Department of infectious diseases University of Missouri‐Kansas City Missouri USA; ^7^ Department of Family Medicine, Hamad General Hospital Hamad Medical Corporation Doha Qatar

**Keywords:** hypoglycaemia, IGF 2, IGF II, insulin‐like growth factor‐2, NICTH, non‐insulin mediated hypoglycaemia, non‐islet cell tumour hypoglycaemia

## Abstract

**Introduction:**

Insulin‐like growth factor‐2 (IGF‐2)‐mediated hypoglycemia is a rare yet clinically significant entity with considerable morbidity and mortality. Existing literature is limited and fails to offer a comprehensive understanding of its clinical trajectory, management and prognostication.

**Methods:**

Systematic review of English‐language articles reporting primary patient data on IMH was searched using electronic databases (PubMed, Scopus and Embase) from any date up to 21 December 2022. Data were analysed in STATA‐16.

**Results:**

The systematic review contains 172 studies, including 1 Randomised controlled trial, 1 prospective observational study, 5 retrospective observational studies, 150 case reports, 11 case series and 4 conference abstracts. A total of 233 patients were analysed, averaging 60.6 ± 17.1 years in age, with comparable proportions of males and females. The commonest tumours associated with Insulin‐like Growth Factor‐2‐mediated hypoglycaemia were fibrous tumours (*N* = 124, 53.2%), followed by non‐fibrous tumours originating from the liver (*N* = 21, 9%), hemangiopericytomas (*N* = 20, 8.5%) and mesotheliomas (*N* = 11, 4.7%). Hypoglycaemia was the presenting feature of NICT in 42% of cases. Predominant clinical features included loss of consciousness (26.7%) and confusion (21%). The mean IGF‐2 and IGF‐1 levels were 882.3 ± 630.6 ng/dL and 41.8 ± 47.8, respectively, with no significant correlation between these levels and patient outcomes. Surgical removal was the most employed treatment modality (47.2%), followed by medication therapy. The recovery rate was 77%, with chronic liver disease (CLD) significantly associated with a poor outcome (OR: 7.23, P: 0.03). Tumours originating from fibrous tissues were significantly associated with recovery (*p* < .001). In the logistic regression model, CLD remained a significant predictor of poor outcomes.

**Conclusion:**

This systematic review highlights that most non‐islet‐cell tumour‐hypoglycaemia (NICTH) is due to fibrous tumours. NICTs demonstrate a variable prognosis, which is fair if originating from fibrous tissue. Management such as octreotide, corticosteroids, diazoxide, embolization, radiotherapy and surgical resection have disparate success rates.

## Graphical abstract

This systematic review explores Insulin‐like Growth Factor‐2 Mediated Hypoglycaemia (IMH) by analysing data from 233 patients. It highlights key insights, such as tumour associations, prognostic indicators (e.g. chronic liver disease) and an 88% positivity rate for IGF2:IGF1 ratio>10. Additionally, IMH‐related tumour mortality can reach 23%, improving treatment and prognosis prediction.

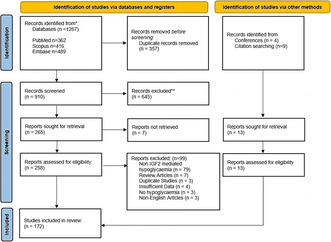

## 1 INTRODUCTION

Hypoglycaemia is commonly defined as a plasma glucose concentration of less than 70 mg/dL, although signs and symptoms may not appear until plasma glucose concentrations fall below 55 mg/dL (3 mmol/L).^1^ Since 1938, the Whipple triad has been used to confirm spontaneous hypoglycaemia before proceeding with further diagnostic workup. Whipple triad criteria include (1) low plasma glucose concentration, (2) signs and symptoms due to hypoglycaemia and (3) remission of the hypoglycaemic symptoms after increasing plasma glucose concentration.^2^, ^3^ According to the current Endocrine Society Clinical Practice Guidelines, hypoglycaemia causes can be divided into two categories based on clinical status: (1) patients who appear to be healthy (endogenous hyperinsulinemia, insulinoma, post‐bariatric hypoglycaemia, autoimmune insulin hypoglycaemia, malicious hypoglycaemia, hypoglycaemia due to antibodies to insulin and insulin receptors), and (2) patients who look to be unwell or medicated (malnutrition, IGF‐2‐secreting non‐islet cell tumour, drugs, alcohol, hypopituitarism and hypoadrenalism).^2^

Hypoglycaemia occurs uncommonly in the non‐diabetic population. Some causes include critical illness, adrenal insufficiency, deficiency of other hormones in glucose metabolism, certain medications (salicylates, sulfa drugs, quinine), stomach surgeries, certain enzyme deficiencies and alcohol intake.^4^ Neoplastic processes within the pancreas can instigate hypoglycaemic conditions by an unchecked overproduction of insulin. Besides these insulinomas or insulin‐secreting tumours within the pancreas, tumours in other body regions may also precipitate hypoglycaemia. These non‐pancreatic tumours induce hypoglycaemia through an unrestrained production of Insulin‐like Growth Factor‐2 (IGF‐2).^4^ IGF‐2 predominantly contributes to embryonic development, functioning as a pivotal regulatory element. However, in adult physiology, IGF‐2 is present in modest quantities, particularly within skeletal muscle tissue, facilitating tissue proliferation. Moreover, IGF‐2 can be detected in increased amounts in injured tissue, where it functions to aid the wound healing process.^5^ IGF‐2 typically demonstrates weak affinity for insulin receptors under normal physiological conditions. However, abnormally elevated levels of IGF‐2 can result in sufficient stimulation of insulin receptors, potentially inducing a state of clinically significant hypoglycaemia. This is mainly observed when IGF‐2 secretion is unchecked, such as in malignancies.^6^

In clinical practice, non‐islet cell tumour hypoglycaemia (NICTH) is considered a rare condition, with an incidence rate of one per million person‐years.^7^, ^8^ The true prevalence and clinical impact of hypoglycaemia induced by NICTH remains uncertain in the current medical literature. However, it is estimated to be four times less prevalent than insulinoma.^9^ NICTH is a paraneoplastic syndrome that manifests through excess IGF‐2‐led insulin receptor activation, causing hypoglycaemia.^7^, ^10^ NICTH was first identified in patients with hepatocellular cancer in 1929. Since then, several different forms of neoplasms have been linked to NICTH. In approximately half of the patients with IGF‐2‐mediated hypoglycaemia, the tumour is identified before developing hypoglycaemia.^11^ In NICTH, workup typically reveals low insulin, beta‐hydroxybutyrate and C‐peptide levels, suggesting an insulin‐mimetic effect in the absence of excess insulin levels, which is an indication to evaluate IGF‐1 and IGF‐2 levels.^12^ A ratio of IGF‐2 and IGF‐1 in a healthy individual is 3:1. A molar ratio larger than 10 in recurrent hypoglycaemia supports NICTH.^7^ Given the relative infrequency of testing for IGF‐1, IGF‐2 and pro‐IGF‐2 testing, the diagnosis of NICTH poses considerable challenges and is often overlooked, which may lead to significant clinical consequences. However, early recognition of NICTH, followed by the implementation of tumour‐targeted and systemic therapeutic approaches, can aid in mitigating its poor prognosis.^13^ Diagnosing paraneoplastic IGF‐2‐induced hypoglycaemia is important and time‐sensitive, as early identification of the underlying tumour permits comprehensive surgical resection. This, in turn, offers the potential for complete resolution and cure of the ensuing hypoglycaemia. A growing body of evidence supports screening for IGF‐2 in non‐diabetic persons with hypoglycaemia and suppressed insulin and c‐peptide levels.^14^

Non‐islet cell tumour hypoglycaemia remains a rare but serious condition with high morbidity and mortality risk. Most literature on NICTH consists of case series and small retrospective studies representing variable results. Current literature is devoid of larger, systematic investigations that thoroughly elucidate the clinical trajectory and prognostic understanding of NICTH. Hence, the prevailing guidelines offer insufficient guidance regarding the optimal therapeutic strategies for managing IGF‐2‐mediated hypoglycaemia. We conducted a systematic review to generate cumulative evidence on the epidemiological characteristics, clinical trajectory, therapeutic strategies and resultant outcomes associated with IGF‐2‐mediated hypoglycaemia.

## 2 METHODS

### 2.1 Literature search

We conducted a systematic literature search on electronic databases (PubMed/Medline, Embase and SCOPUS) from any date to 12 December 2022, for English‐language articles on IGF‐2‐mediated hypoglycaemia. Keywords and mesh terms used to create search syntax included: “IGF 2” OR “IGF II” OR “insulin‐like growth factor” AND “Non‐islet Cell Hypoglycaemia” OR “NICH” OR “Non‐islet‐cell tumour hypoglycaemia” OR “NICTH” OR “Hypoglycaemia” OR “Neuroglycopenia” OR “Glucose” [All Fields].

### 2.2 Study selection

The articles were exported from the databases to endnote 9 and then transferred to Rayyan AI for screening. HC and AAK screened the retrieved articles independently, initially from the title and abstract, followed by full‐text review of the shortlisted studies. FA reviewed the disputed articles independently to resolve conflicts.

### 2.3 Inclusion criteria

The inclusion criteria were English‐language original studies reporting primary data (case reports, series, observational retrospective, prospective studies and clinical trials) regarding IGF‐2‐mediated hypoglycaemia.

### 2.4 Exclusion criteria

Studies reporting IGF‐1 as a cause of hypoglycaemia or studies mentioning causes of hypoglycaemia other than IGF‐2 were excluded from the review. Studies reporting high IGF‐2 levels without hypoglycaemia were also excluded. Animal studies on IGF‐2‐mediated hypoglycaemia were also excluded from this review. Finally, studies reporting secondary patient data were also excluded.

### 2.5 Quality assessment

The AAK and IK performed quality assessment of the included studies. Case reports and series were assessed using the Joanna Briggs Institute case report appraisal checklist for inclusion in systematic reviews.^15^ Observational studies were assessed using the methodological index for non‐randomised studies (MINORS) scoring system.^16^ Finally, the quality of clinical trials was assessed using the Cochrane Collaboration's tool.^17^ FA resolved the conflicts via an independent quality assessment in case of disagreements.

### 2.6 Data collection and statistical analyses

Four reviewers collected baseline demographic details, including age, gender and comorbidities of the patients. Data were double‐checked for accuracy by two reviewers who were not initially involved in the data collection. Symptoms of hypoglycaemia were collected. The data of the initial hypoglycaemia workup was collected, including glucose levels with corresponding insulin (μIU/mL), C‐peptide (ng/mL), beta‐hydroxybutyrate (mmol/L), pro‐insulin, oral hypoglycaemic screen and insulin antibody results. The lowest pre‐treatment glucose level was also extracted. All data was collected and reported where available. We collected the highest pre‐treatment levels of IGF‐1 (ng/mL), IGF‐2 (ng/mL) and IGF binding protein (IGFBP). Data on big IGF‐2 molecules was also collected. Tumour‐related data included the type, location and size of tumours, radiological details, biopsy and histopathology findings of the tumours. We encountered a diverse range of cancers/tumours, necessitating a categorization approach for analysis. Due to the wide variety of tumour types, it was impractical to analyse and summarise each individually. Therefore, we classified them into two broad categories: fibrous and non‐fibrous tumours. The fibrous category encompassed numerous fibromatous tumours, regardless of the region. Non‐fibrous tumours included primary cancers or tumours based on the region. Treatment data included surgery, embolisation, radiotherapy, steroid use and other medical management. Outcome data included tumour recurrence, hypoglycaemia and in‐hospital mortality. No imputation was made to address the missing data. We followed the Preferred Reporting Items for Systematic Reviews and Meta‐Analyses (PRISMA) guidelines for reporting systematic reviews in this study.^18^ Data were analysed using R Studio version 2023.03.0 (build 386). The Mann–Whitney *U* test and Chi‐square test were used as appropriate. A logistic regression model was also developed, as discussed in the results section. We reported results as means with standard deviation, medians with interquartile ranges, or numbers with percentages as appropriate.

## 3 RESULTS

**FIGURE 1:**
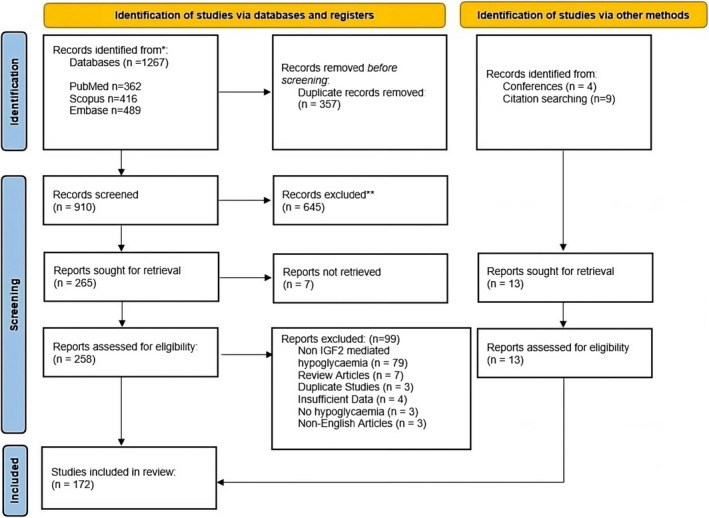
Prisma flow chart of the screening process of eligible studies with details of included and excluded articles.

A total of 172 studies were included in this systematic review (Figure 1). These included one randomised controlled trial, one prospective observational study, five retrospective observational studies, 150 case reports, 11 case series and four conference abstracts (Data S1).

### 3.1 Baseline characteristics

A total of 233 participants were identified, with 125 males and 108 females. The mean age was 60.6 ± 17.1 years. Hypoglycaemia was identified as the presenting feature of NICT in 42% (98) of patients. Loss of consciousness and confusion were the most common features in the patients, present in 26.7% and 21%, respectively. Weight loss, dizziness and weakness were other relatively less common features. Hypertension and diabetes were the two most common comorbidities prevalent in 11.6% and 6.9% of patients, respectively. Chronic liver disease (CLD), heart failure and chronic kidney disease were found in 2.6%, 2.1% and 1.3% of patients.

### 3.2 Etiologies and presenting features of hypoglycaemia

**FIGURE 2:**
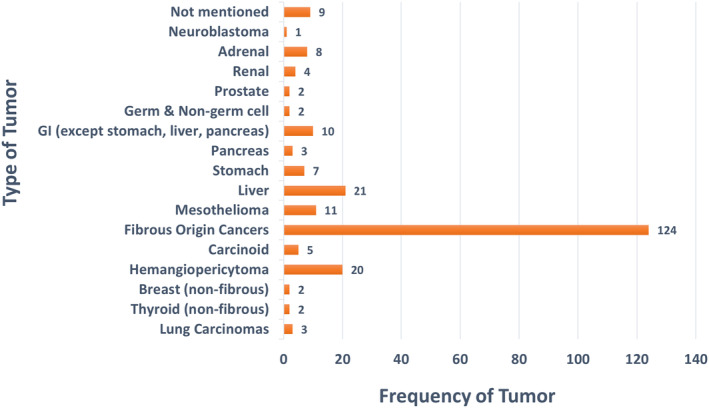
Types and frequencies of tumours associated with IGF‐2‐mediated hypoglycaemia (*N* = 233).

The most common tumours associated with IGF‐2‐mediated hypoglycaemia were fibrous tumours (*N* = 124, 53.2%), followed by non‐fibrous tumours originating from the liver (*N* = 21, 9%), hemangiopericytoma (*N* = 20, 8.5%) and mesothelium (*N* = 11, 4.7%). Tumour type and origin were not specified in 9 patients (3.9%). The anatomical data of tumours is presented in Figure 2.

### 3.3 Diagnosis of IGF‐II mediated hypoglycaemia

**FIGURE 3:**
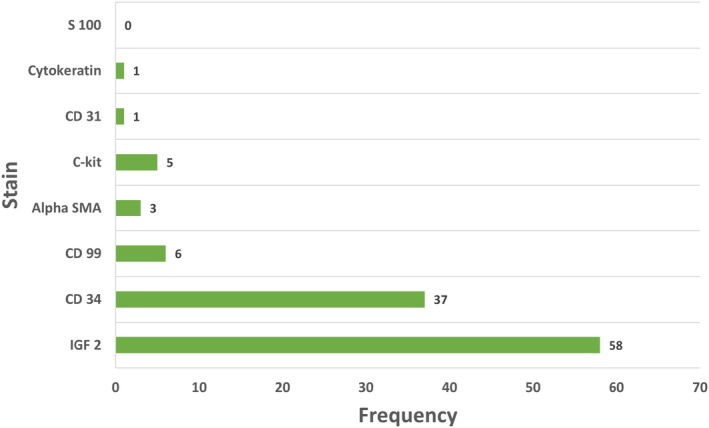
Type and frequency of various histological stains reported in patients with IGF‐2‐mediated hypoglycaemia (*N* = 88).

The average IGF2 and IGF1 levels in this study were 882.3 ± 630.6 ng/dL and 41.8 ± 47.8, respectively. The IGF 2:1 ratio was 192.8 +/− 1704.9, but this was varied due to three outlier values above 500. They were 500.7, 6670 and 22,150 in 3 cases. Excluding these three outliers, the average ratio was 32.3 ± 36.3 (Table 1). Cancers originating from fibrous tissues (fibroma and fibrosarcoma) were the most associated cancers, comprising 123 cases, with gastrointestinal cancers being the second most common. Hemangiopericytoma cases comprised another 20 (8.5%) cases in our cohort. Histological data regarding tumour staining was available from 88 patients (37.7%). Staining was reported for CD34, CD31, CD99, S100 protein, IGF‐2, cytokeratin, C‐kit and alpha‐SMA. The relevant data is reported in Figure 3.

**TABLE 1:** Baseline clinical characteristics of patients with IGF‐2 mediated hypoglycaemia (*N* = 233).

Characteristic	Statistic
Age in years	Mean: 60.6
	SD: 17.1
Gender	Male: 125 (53.6%)
	Females: 108 (46.3%)
Asymptomatic presentation	3/233 (1.3%)
Hypoglycaemia	97/233 (41.6%)
Syncope	24/233 (10.3%)
Dizziness	21/233 (9.0%)
Loss of consciousness	62/233 (26.7%)
Palpitations	8/233 (3.4%)
Visual disturbances	5/233 (2.1%)
Weight loss	20/233 (8.6%)
weight gain	2/233 (0.8%)
Weakness	25/233 (10.7%)
Confusion	49/233 (21%)
Focal signs	9/233 (3.9%)
Seizures	11/233 (4.7%)
Diabetes	16/233 (6.9%)
Hypertension	27/233 (11.5%)
Heart failure	5/233 (2.1%)
Chronic liver disease	6/233 (2.6%)
Chronic kidney disease	3/233 (1.3%)
Coronary artery disease	2/233 (0.8%)
Hypothyroidism	4/233 (1.7%)
Hyperthyroidism	2/233 (0.8%)
Psychiatric disease	1/233 (0.4%)
PET	18/233 (7.8%)
CT Thorax	69/233 (29.6%)
CT Abdomen	27/233 (11.6%)
IGF1 (ng/ml)	Mean: 41.8
	Std. Dev:47.8
	Median: 27
	IQR:20.6–52
IGF2 (ng/ml)	Mean: 882.3
	Std. Dev: 630.6
	Median: 763.4
	IQR: 489.3–1088.1
IGF (2:1) Ratio	Median: 13.3
	IQR: 13.3–43.2
Treatments employed	Surgical Removal: 110 (47.2%)
	Embolization: 14 (6.0%)
	Radiotherapy: 14 (6.0%)
	Steroids: 91 (39.1%)
	Diazoxide: 16 (6.9%)
	Octreotide: 18 (7.7%)

*Note*: Data presented as mean (SD), median (IQR) and numbers (%) as appropriate.

Abbreviations: IQR: Interquartile range; Std. Dev, Standard Deviation.

### 3.4 Diagnostic testing and treatment details

Surgical removal was the most common treatment modality employed in 110 (47.2%) cases. Embolisation and radiotherapy were used in 14 (6%) cases each. Medication therapy with corticosteroids, octreotide and diazoxide was used in 91 (39.1%), 18 (7.7%) and 16 (6.9%) cases, respectively. The type of steroids was specified in 74 cases. Most of the patients were started on prednisone (*N* = 55, 74.3%), followed by dexamethasone (*N* = 13, 17.6%), hydrocortisone (*N* = 5, 6.7%) and methylprednisolone (*N* = 1, 1.3%). The dose of steroids was reported in 65 cases. The mean initial prednisone daily equivalent dose was 30 mg/day (20–20).

### 3.5 Outcome analysis

Outcomes were available for 223 cases, and the overall recovery rate among all the participants was 77% (*n* = 172). There was no significant difference between recovered and non‐recovered patients regarding gender, age, clinical presentations, or comorbidities except for chronic liver disease **(**Table 2 **).** Chronic liver disease (CLD) was more likely to be associated with death than recovery (OR: 7.23, P: 0.03). Of the 233 patients, only six had CLD. These patients had hepatocellular carcinoma (*N* = 4), gastrointestinal stromal tumour (*N* = 1) and metastatic hemangiopericytoma (*N* = 1). IGF values at presentation or IGF 2:1 ratio were not associated with any outcome, as shown in Table 2. Fibrous tumours were significantly associated with recovery compared to non‐fibrous ones, and the association was statistically significant (65.1% vs. 33%, *p* < .001).

**TABLE 2:** Outcome analysis (based on mortality vs. recovery) among patients with IGF‐2‐mediated hypoglycaemia.

Characteristics		Recovered (*n* = 172)	Died (*n* = 51)	*p*‐Value
	Age	Median: 63	Median: 60	.18
		IQR: 50–75	IQR: 46–69.5	
	Gender	Male: 93 (54.1%)	Males: 27 (52.9%)	1
		Females: 79 (45.9%)	Female: 24 (47.1%)	
Presentation	Asymptomatic Presentation	2 (1.2%)	1 (1.9%)	1
	Hypoglycaemia	82 (47.7%)	15 (29%)	1
	Syncope	19 (11.0%)	5 (9.8%)	1
	Dizziness	16 (31.4%)	5 (9.8%)	1
	Loss of Consciousness	49 (28.5%)	13 (25.5%)	.8
	Palpitations	6 (3.5%)	2 (3.9%)	1
	Visual Disturbances	3 (1.7%)	2 (3.9%)	.7
	Weight Loss	14 (8.1%)	6 (11.8%)	.6
	Weight Gain	2 (1.2%)	0	1
	Weakness	16 (9.3%)	9 (17.6%)	.15
	Confusion	37 (21.5%)	12 (23.5%)	.91
	Focal Signs	5 (2.9%)	4 (7.8%)	.24
	Seizures	10 (5.8%)	1 (1.9%)	.45
Comorbidities	Diabetes	13 (7.6%)	3 (5.9%)	.9
	Hypertension	22 (12.8%)	5 (9.8%)	.74
	Heart Failure	4 (2.3%)	1 (1.9%)	1
	Chronic Liver Disease	2 (1.2%)	4 (7.8%)	**.036**
	Chronic Kidney Disease	3 (1.7%)	0	.79
	Coronary Artery Disease	2 (1.2%)	0	1
	Hypothyroidism	3 (1.7%)	1 (1.9%)	1
	Hyperthyroidism	2 (1.2%)	0	1
	Adrenal Insufficiency	0	0	–
	Psychiatric Disease	1 (0.6%)	0	–
	Tumour	172 (100%)	51 (100%)	–
	Fibrous origin versus non‐fibrous tumours	112 (65.1%)	17 (33.3%)	**<.001**
Diagnostic Testing	IGF1	Median: 29.4	Median: 25.6	.1
		IQR: 20.7–55.5	IQR: 16.6–32.6	
	IGF2	Median: 767.7	Median: 667	.4
		IQR: 501.9–1124.7	IQR: 473–982.1	
	IGF 2: 1 Ratio^a^	Median: 20.4	Median:23.0	.19
		IQR: 11.8–37.3	IQR: 17.6–42.9	
	Insulin Ab	2 (1.2%)	0	–
	PET	11 (6.4%)	7 (13.7%)	.16
	CT Thorax	57 (33.1%)	12 (23.5%)	.25
	CT Abdomen	72 (41.9%)	28 (54.9%)	.13
Treatments	Surgery	95 (55.2%)	15 (29.4%)	**.002**
	Embolization	9 (5.2%)	5 (9.8%)	.39
	Radiotherapy	9 (5.2%)	5 (9.8%)	.39
	Steroids	60 (34.9%)	31 (60.8%)	**.001**
	Diazoxide	10 (5.8%)	6 (11.8%)	.25
	Octreotide	9 (5.2%)	9 (17.6%)	**.01**

*Note*: Data presented as mean (SD), median (IQR) and numbers (%) as appropriate. *p* values in bold are statistically significant.

Abbreviations: IGF, Insulin like growth factor; IQR, Interquartile range, Std. Dev, Standard Deviation.

^a^ The values represent the ones after excluding the outliers.

### 3.6 Comparison of IGF 2:1 ratio

Another analysis with the IGF 2:1 ratio used as a dependent variable was done to see if it was associated with our study variables (Table 3). Overall, the IGF 2:1 ratio was not significantly different between genders, presentations, comorbidities, or treatment groups. However, patients with hypoglycaemia as the first presentation of NICT had a lower IGF 2:1 ratio than those without (Median: 18.9 vs. 23.4), and the association was statistically significant (*p* = .046).

**TABLE 3:** Comparison of Median IGF (2:1) Ratio based on presence or absence of clinical variables among the patients with reported IGF 1 and 2 levels.

	Variable	Present	Absent	*p*‐Value
Presentations	Asymptomatic Presentation	Median: 14.8	Median: 22.5	.46
		IQR: 14.8–14.8	IQR: 13.3–42.9	
	Hypoglycaemia as the first presentation	Median: 18.9	Median: 23.4	**.046**
		IQR: 11.8–31.9	IQR:14.5–45.8	
	Syncope	Median: 17.4	Median: 22.5	.41
		IQR: 12.6–28.6	IQR: 13.6–44.0	
	Dizziness	Median: 22.2	Median: 22.5	.12
		IQR 9.13–18.7	IQR 14–44.6	
	Loss of Consciousness	Median: 20.4	Median: 22.5	.76
		IQR: 12.4–45.9	IQR: 14–40.75	
	Palpitations	Median:27.7	Median:22.1	.47
		IQR: 18.7–69.9	IQR: 13.6–42.2	
	Visual Disturbances	Median: 21.9	Median: 22	.82
		IQR: 16–24.3	IQR: 13.3–42.9	
	Weakness	Median: 20.4	Median: 22.4	.97
		IQR: 13.9–1410.6	IQR: 13.3–42.6	
	Confusion	Median: 21.7	Median: 22.9	.46
		IQR: 15.7–28.4	IQR: 13.3–44.1	
	Focal Signs	Median: 16.7	Median: 22.9	.15
		IQR: 13.9–17.8	IQR: 13.5–43.7	
	Seizures	Median: 22.9	Median: 22.2	.63
		IQR: 18.2–25.4	IQR: 13.3–44	
Comorbidities	Diabetes	Median: 15.8	Median: 22.7	.27
		IQR: 10.2–29	IQR: 13.9–42.5	
	Hypertension	Median: 19.1	Median: 22.7	.42
		IQR: 11.1–34.9	IQR: 14–43.7	
	Heart Failure	Median: 54.6	Median: 22.1	.11
		IQR: 13.3–40	IQR: 43.9–56.7	
	Chronic Liver Disease	Median: 33.3	Median: 22.3	.41
		IQR: 20.2–49.9	IQR: 13.3–41.1	
	Chronic Kidney Disease	Median: 11.3	Median: 22.5	.63
		IQR: 11.3–35.2	IQR: 13.8–42.2	
	Coronary Artery Disease	Median: 17.2	Median: 22.5	.6
		IQR: 17.2–17.2	IQR: 13.3–42.9	
	Hypothyroidism	Median: 18.5	Median: 22.5	.66
		IQR: 18.0–64.1	IQR: 13.3–42.2	
	Hyperthyroidism	9.7	Median: 22.5	.22
		IQR: 9.7–9.7	IQR: 13.8–42.9	
	Psychiatric Disease	20.4	Median: 22.5	.84
		IQR: 20.4–20.4	IQR: 13.4–42.9	
	Fibrous Vs Non‐Fibrous Tumours	34.6	Median: 22.13	**.001**
		IQR: 20.4–45.2	IQR: 3–40.1	
Diagnostic Testing	PET	34.6	Median: 22.13	**<.001**
		IQR: 20.4–45.2	IQR: 3–40.1	
	CT Thorax	18.2	Median: 23.4	**.012**
		IQR: 10.5–26.7	IQR: 14.5–45.1	
	CT Abdo	20.7	Median: 23.0	.34
		IQR: 11.3–34.6	IQR: 14–44.1	
Treatments	Surgery	Median: 20.3	Median: 23.2	.25
		IQR: 11.3–39.6	IQR: 14.8–42.9	
	Embolization	Median: 24.1	Median: 22.2	.81
		IQR: 13.641.3	IQR: 13.6–42.2	
	Radiotherapy	Median: 23.6	Median: 22.1	.43
		IQR: 19.1–40.7	IQR: 13.3–41.8	
	Steroids	Median: 22.14	Median: 22.7	.87
		IQR: 14.8–33.1	IQR: 12.3–45.1	
	Diazoxide	Median: 23.5	Median: 22.2	.91
		IQR: 16.1–24.1	IQR: 13.2–43.5	
	Octreotide	Median: 22.9	Median: 22.4	.50
		IQR: 14.8–48.3	IQR: 13.2–41.1	

*Note*: *p* values represent Man‐Whitney *U* test. *p* values in bold are statistically significant.

### 3.7 Logistic regression outcome model

A logistic regression model with the outcome as a dependent variable was developed using the IGF 2:1 ratio, tumour types and chronic liver disease as independent variables (Table 4). Tumour category and chronic liver disease were still significant predictors of outcome while adjusting each other and for IGF 2:1 ratio (*p* = .001 and *p* = .03, respectively) as seen in Table 4.

**TABLE 4:** Multivariate logistic regression model for outcome analysis.

Variable	Odd's ratio (95% CI)	*p*‐Value
IGF 2:1 ratio	0.99 (0.99–1.00)	.83
Tumour category (Non‐Fibrous vs. Fibrous)	1.23 (1.09–1.39)	<.001*
Chronic liver disease	0.65 (0.44–0.96)	.03*

*Note*: Adjusted for IGF 2:1 ratio. * signifies values that remained statistically significant after multivariate regression.

Abbreviation: CI, Confidence Interval.

## 4 DISCUSSION

This systematic review focuses on IGF‐2‐mediated hypoglycaemia, encompassing a wide range of underlying tumours. Of the 233 patients, most had an underlying fibrous tumour (53.2%). Generally, the patients were older adults (mean age 60.6 years). After excluding the outliers, the mean IGF‐2:IGF‐1 ratio was 34.9 ng/dL. Surgical intervention emerged as the most prevalent treatment modality, employed in 47.2% of cases, followed by steroid therapy (39.1%). Other treatments included embolisation, radiotherapy and other drugs such as octreotide and diazoxide. Most patients successfully recovered (77%); however, a significant mortality rate was identified (23%). Multivariate logistic regression analysis identified chronic liver disease as a significant predictor of adverse outcomes, while a fibrous origin of the tumour was associated with a more favourable prognosis in patients with IGF‐2‐mediated hypoglycaemia.

A complex interplay of multiple mechanisms underlies the pathophysiology of hypoglycaemia in patients with NICT. The primary driver is the tumours' excessive production of immature and mature IGF‐2 molecules.^7^ Other mechanisms that can play an essential role in the development of clinically significant hypoglycaemia include increased uptake of glucose by the tumour cells (especially if of muscle origin) and replacement of liver tissue responsible for as a presenting feature.^11^ Irrespective of the underlying etiological mechanisms, hypoglycaemia stands as the most clinically salient manifestation of these tumours. This necessitates concentrated research efforts to elucidate optimal management guidelines for managing general and cause‐specific hypoglycaemia. A diverse array of anatomical and pathological tumour types has been implicated in the aetiology of IGF‐2‐mediated hypoglycaemia, with frequencies varying considerably across existing literature. In a retrospective study on 78 patients with NICTH, Fukuda et al. reported the highest prevalence of liver and gastric malignancies.^19^ The study was not included in this systematic review due to insufficient data. While the mean age of the patient cohort in our analysis aligns with that reported in the retrospective study, we observed significant disparities, most notably in the predominant malignancies causing NICTH. Our study identified fibrous tumours as the leading aetiology, accounting for 53.2% of NICTH cases. In contrast, the study by Fukuda et al. reported a markedly lower prevalence, with only 5% of the tumours being of fibrous origin. However, older reviews have documented fibromas as one of the most common aetiologies of NICTH.^20^ Historically, discrepancies in the literature have been attributed to small sample sizes, introducing potential bias. With an evaluation of 233 patients, our study constitutes the most extensive systematic dataset to date, thereby corroborating and solidifying prior findings regarding the most prevalent tumour types associated with non‐islet cell tumour hypoglycaemia (NICTH).

The diagnosis of IGF‐2‐mediated mechanism behind NICTH is sometimes complex and needs careful evaluation and ruling out other more common causes of hypoglycaemia. A raised IGF‐2 to IGF‐1 ratio from a normal 3:1 to 10:1 has been referred to confirmation for NICTH secondary to IGF‐2 action.^7^ Our study corroborates this observation, revealing a ratio of IGF‐2 to IGF‐1 exceeding 10 in 87.6% of the patient cohort. In diagnostically challenging scenarios, this elevated ratio is a valuable confirmatory marker, facilitating timely clinical management. Nevertheless, a low ratio does not effectively rule out IGF‐2 mediation as sometimes abnormal IGF‐2 molecules may not be detected in laboratory assays and may cause a diagnostic bias.^6^

Additionally, the ratio of IGF‐2:IGF:1 is influenced by the presence of IGF‐binding proteins (IGFBP). High levels of IGFBP can cause falsely low ratios.^6^ In our study, we found a lower IGF‐2:IGF‐1 ratio in patients presenting with hypoglycaemia as the first manifestation of NICTH. This may be attributed to multiple interrelated factors. Firstly, aberrant processing of pro‐IGF‐2 in certain tumours leads to increased levels of ‘big’ IGF‐2, which have less efficacy to bind to IGF receptors, thereby potentially lowering the calculated IGF‐2:IGF‐1 ratio.^21^, ^22^ Secondly, individual variability in receptor sensitivity to IGF‐2 might influence the clinical manifestation of hypoglycaemia, independent of the IGF‐2:IGF‐1 ratio. Thirdly, alterations in IGF binding proteins (IGFBPs), which regulate IGF‐2's bioavailability and activity, can impact the observed ratio.^21^, ^22^ Additionally, tumour heterogeneity contributes to the variance in IGF‐2 secretion and metabolic impact, reflecting in the IGF‐2:IGF‐1 ratio.^21^, ^22^ Finally, dynamic and adaptive endocrine feedback mechanisms could affect this ratio. In the context of IGF‐2‐mediated hypoglycaemia, the hypothalamic–pituitary axis might respond to the elevated levels of IGF‐2 (and its resultant effects on blood glucose levels) by adjusting the secretion of other hormones. These changes can alter the insulin sensitivity, glucose metabolism and the overall balance between IGF‐2 and IGF‐1.^11^

Histological staining of tumours is an under‐reported data in studies on IGF‐2‐mediated hypoglycaemia. Case reports have shown various stains positive in these tumours.^6^ Our study provides the largest cumulative evidence of stain patterns in the patients. IGF‐2 stain was positive in most patients where data was available (65.9%), followed by CD 34 (42%). The diagnostic and prognostic significance of histologic staining remains unexplored and is limited due to the scarcity of dedicated studies. Another limitation is the availability of staining in the diagnostic centres.

Nonetheless, as additional data on tumour staining techniques accumulate and as these methods become increasingly accessible in diagnostic centres, their utility in diagnosing and clinically managing NICTH for complex cases may be of considerable significance. Interestingly, our analysis shows that higher levels of IGF2 alone might not be clinically significant when reviewed alone. Our analysis of the IGF‐2 to IGF‐1 ratio disclosed lower ratios in patients for whom hypoglycaemia constituted the initial clinical presentation. Moreover, a multivariate logistic regression model evaluating outcomes failed to identify IGF‐2 levels as a statistically significant predictor of mortality. This highlights the imperative of a holistic evaluation of individual cases when formulating subsequent management strategies. While IGF levels may aid in the diagnosis of complex cases, our findings indicate that prognostic assessments cannot be predicated solely upon these biochemical markers.

Previous investigations involving smaller cohorts have posited that approximately half of the patients with NICT may present with hypoglycaemia as an initial clinical manifestation.^6^, ^19^ The current investigation also shows that a sizable proportion (42%) of patients had hypoglycaemia at the initial presentation. Nonetheless, the prognostic implications of hypoglycaemia as an initial clinical presentation remain ambiguous and warrant further prospective evaluation to ascertain its prognosis. The treatment of IGF‐2‐mediated hypoglycaemia can be divided into two phases: acute management, which can be similar to hypoglycaemia of other causes, and definitive treatment by focusing on reduction in tumour function. Bodnar et al.^21^ generated a narrative review focused on the management of NICTH in 2014. The treatment options reported by Bodnar et al. persist, and we did not find any new management strategy in the current study. The selection of therapeutic modalities ranging from surgical intervention to localised tumour‐targeting approaches such as embolisation and radiotherapy, as well as pharmacological agents including corticosteroids, diazoxide, growth hormone and octreotide, is principally guided by the type, anatomical location and extent of the tumour. A multimodal management approach is sometimes required, including specialised and multidisciplinary care.^23^ In the management of IGF2‐mediated hypoglycaemia, particularly in challenging cases of tumour‐induced hypoglycaemia unresponsive to conventional therapies, Pasireotide might be a future option once more evidence in its broader efficacy is available.^24^ This second‐generation somatostatin receptor analogue, with its enhanced affinity for somatostatin receptor subtype 5 (SSTR5), effectively inhibits excessive insulin secretion, one of the factors in insulinoma and NICTH.^25^, ^26^ Evidence regarding its use in NICTH is extremely limited, although it is plausible due to its potential to suppress IGF‐2. Pasireotide ‘s dual anti‐hypoglycaemic and antitumor properties may make it a potentially favourable first‐line treatment option, especially when surgical interventions are not possible or other medical treatments prove ineffective.^24^

The literature lacks studies investigating factors associated with mortality in patients with IGF‐2‐mediated hypoglycaemia. Multivariate logistic regression analysis in our study indicated that fibrous tumours are associated with a more favourable prognosis in terms of mortality outcomes. This aligns with the general understanding of fibrous tumours, which have a better prognosis than other types of tumours.^27^ Furthermore, our analysis identified CLD as a significant predictor of mortality. The occurrence of hypoglycaemia in the context of CLD is an established determinant of adverse outcomes.^28^ However, how CLD can worsen prognosis in patients with IGF‐2‐mediated hypoglycaemia is not studied. As gluconeogenesis and glycogenolysis can be compromised in patients with CLD, the effects of IGF‐2‐mediated hypoglycaemia can potentiate and might lead to severe manifestations and worse outcomes.^29^ In light of a high mortality rate, approximating 23%, identifying and comprehensively evaluating prognostic factors is paramount and necessitate further investigative efforts.

### 4.1 Strengths and limitations

The primary strength of this investigation lies in its comprehensive, systematic appraisal of data pertaining to IGF‐2‐mediated hypoglycaemia. The infrequent incidence of non‐islet cell tumour hypoglycaemia poses challenges to the execution of large‐scale, prospective study designs, further underscoring the significance of our work. Consequently, the synthesis of previously published data in our study enhances the understanding of this phenomenon, providing a clearer, more rigorous framework for both clinical interpretation and future research endeavours. Nevertheless, it is imperative to acknowledge the limitations inherent to this study, which predominantly stem from conducting a systematic review based on observational data. This review included only those patients with NICT who developed hypoglycaemia; hence, the prevalence or burden of NICT as a whole could not be calculated. Secondly, the potential impact of publication bias must be acknowledged, given that unpublished studies could have influenced the aggregated findings of this systematic review. Furthermore, the exclusion of certain studies from this review due to the unavailability of individual‐level data constitutes an additional limitation that could potentially impact the comprehensiveness and generalizability of our findings. Nevertheless, this review highlights the clinical trajectory of IGF‐2‐mediated hypoglycaemia and identifies key prognostic factors. It serves as a foundational resource, paving the way for future research to identify factors that could enhance diagnostic accuracy and therapeutic management.

## 5 CONCLUSION

IGF‐2‐mediated hypoglycaemia is a rare manifestation of non‐islet cell tumours. This elaborative systematic review highlights that most non‐islet cell tumours hypoglycaemia (NICTH) is due to fibrous tumours. Furthermore, NICTH‐related tumours demonstrate a variable prognosis, which is fair if they originate from fibrous tissue. A notable mortality rate, approximating one‐fourth of the patients, highlights significant healthcare implications and necessitates urgent attention to clinical management strategies. Various therapeutic modalities have been employed, encompassing pharmacological interventions such as octreotide, corticosteroids and diazoxide, as well as embolization, radiotherapy and surgical resection, with variable success rates. The inherent rarity of IGF2‐mediated hypoglycaemia presents unique challenges for traditional prospective research methodologies needed to generate high‐level evidence. Recognising this, it is more practical to utilise tailored statistical approaches tailored to rare disease research, such as case–control studies, with an in‐depth investigation of fewer cases. Additionally, utilising patient registries and real‐world data may provide valuable longitudinal information.

## AUTHOR CONTRIBUTIONS

**Fateen Ata:** Conceptualization (lead); data curation (lead); formal analysis (lead); funding acquisition (lead); investigation (lead); methodology (lead); project administration (lead); resources (lead); supervision (lead); validation (lead); writing – original draft (lead); writing – review and editing (lead). **Hassan Choudry:** Data curation (equal); formal analysis (equal); investigation (equal); methodology (supporting); writing – original draft (supporting); writing – review and editing (supporting). **Adeel Ahmad Khan:** Data curation (supporting); formal analysis (equal); investigation (equal); methodology (equal); writing – original draft (supporting); writing – review and editing (supporting). **Anum:** Data curation (equal); investigation (equal); methodology (supporting); writing – original draft (supporting). **Ibrahim Khamees:** Data curation (equal); investigation (equal); writing – original draft (supporting). **Anas Al‐sadi:** Data curation (equal); investigation (equal); writing – original draft (supporting). **Abdelaziz Mohamed:** Data curation (equal); investigation (supporting); writing – original draft (supporting). **Lujain Malkawi:** Data curation (equal); investigation (supporting); writing – original draft (supporting). **Esra'a Aljaloudi:** Data curation (equal); investigation (supporting); writing – original draft (supporting).

## FUNDING INFORMATION

This study was not funded.

## CONFLICT OF INTEREST STATEMENT

The authors declare that they have no competing interests.

## ETHICS STATEMENT

Private information from individuals will not be published. This systematic review also does not involve endangering participant rights. Ethical approval was not required.

## REGISTRATION

The systematic review is registered with the International Prospective Register of Systematic Reviews (PROSPERO) with the identification number CRD42022381708 and can be found on the given link: https://www.crd.york.ac.uk/PROSPERO/display_record.php?RecordID=381708.

## NOVELTY

Our study not only advances the current understanding of IGF‐2 mediated hypoglycaemia (IMH) but also introduces a novel perspective by unveiling statistically significant prognostic indicators and a high positivity rate for the IGF2:IGF1 ratio. These findings represent a significant step forward in the field, offering new avenues for precision medicine and enhanced patient care.

## IMPORTANCE OF THE STUDY

We present a comprehensive systematic review that fills critical gaps in the understanding of IMH. By analysing data from 233 patients, our review presents novel epidemiological insights, sheds light on tumour types commonly associated with IMH, and offers statistically significant prognostic indicators like chronic liver disease and fibrous origin of tumours. We identified that IGF2:IGF1 ratio>10 has around 88% positivity rate. Our findings showed that mortality rate in IMH‐related tumours can be up to 23%. This study not only refines our comprehension of IMH but also paves the way for a more targeted approach in its treatment and prognosis prediction.

## Supporting information


Data S1.

## Data Availability

Data can be shared on a reasonable request.
