# Supplementary material for: A systematic review of literature on Insulin‐like growth factor‐2‐mediated hypoglycaemia in non‐islet cell tumours
Source: Endocrinol Diabetes Metab. 2024 Feb 27;7(2):e00471. doi: 10.1002/edm2.471 (PMC10897872; doi:10.1002/edm2.471)
Supplement: Supplementary file 1 — Data S1. [file EDM2-7-e00471-s001.pdf]

Studies included in the systematic review [1-172]:

1. Agus MSD, Katz LEL, Satin-Smith M, Meadows AT, Hintz RL, Cohen P. Non-islet-cell tumor associated with hypoglycemia in a child: Successful long-term therapy with growth hormone. *Journal of Pediatrics.* 1995;127(3):403-407.
2. Alkemade GM, Bakker M, Rikhof B, et al. Hypoglycemia in a patient with a big "big"-IGF-II-producing tumor. *J Clin Endocrinol Metab.* 2013;98(8):3113-3114.
3. Almaghraby A, Brickman WJ, Goldstein JA, Habiby RL. Refractory hypoglycemia in a pediatric patient with desmoplastic small round cell tumor. *Journal of Pediatric Endocrinology and Metabolism.* 2018;31(8):947-950.
4. Ang N, Carelli MG, Ramponi F, Saxena P. Case Report: An Interesting Presentation of Hypoglycaemia. *Heart Lung and Circulation.* 2021;30(Supplement 1):S46-S47.
5. Baig M, Hintz RL, Baker BK, Vesely DL. Hypoglycemia attributable to insulin-like growth factor-II prohormone-producing metastatic leiomyosarcoma. *Endocr Pract.* 1999;5(1):37-42.
6. Barra WF, Castro G, Hoff AO, Siqueira SA, Hoff PM. Symptomatic hypoglycemia related to inappropriately high igf-ii serum levels in a patient with desmoplastic small round cell tumor. *Case Rep Med.* 2010;2010:684045.
7. Baxter RC, Holman SR, Corbould A, Stranks S, Jean Ho P, Braund W. Regulation of the insulin-like growth factors and their binding proteins by glucocorticoid and growth hormone in nonislet cell tumor hypoglycemia. *Journal of Clinical Endocrinology and Metabolism.* 1995;80(9):2700-2708.
8. Behringer-Massera S, Brutsaert EF, Epstein EJ. Refractory Hypoglycemia from Paraneoplastic Insulin-Like growth Factor 2 Secretion in A Patient with Hepatocellular Carcinoma. *AACE Clin Case Rep.* 2017;3(4):364-366.
9. Berman J, Harland S. Hypoglycaemia caused by secretion of insulin-like growth factor 2 in a primary renal cell carcinoma. *Clinical Oncology.* 2001;13(5):367-369.
10. Bessell EM, Selby C, Ellis IO. Severe hypoglycaemia caused by raised insulin-like growth factor II in disseminated breast cancer. *J Clin Pathol.* 1999;52(10):780-781.
11. Bourcigaux N, Arnault-Ouary G, Christol R, Perin L, Charbonnel B, Le Bouc Y. Treatment of hypoglycemia using combined glucocorticoid and recombinant human growth hormone in a patient with a metastatic non-islet cell tumor hypoglycemia. *Clinical Therapeutics.* 2005;27(2):246-251.
12. Bruno MC, Moreira MLM, Delai A, et al. Rare case of tumor producer of IGF-II causing hypoglycemia in patient with meningeal hemangiopericytoma. *Diabetology and Metabolic Syndrome Conference: 21st Brazilian Diabetes Society Congress Sao Paulo Brazil.* 2018;10(Supplement 1).
13. Buras ED, Weatherup E, Wyckoff J. Subcutaneous glucagon infusion and continuous glucose monitoring enable effective management of hypoglycemia in a patient with IGF-2-producing hemangiopericytoma. *Clin Diabetes Endocrinol.* 2018;4:2.
14. Carpentier M, Van Huffel L, Lapauw B. Hypoglycemia due to big IGF-2. *Acta Clinica Belgica: International Journal of Clinical and Laboratory Medicine.* 2016;71(Supplement 2):3-4.
15. Chan G, Horton PJ, Thyssen S, et al. Malignant transformation of a solitary fibrous tumor of the liver and intractable hypoglycemia. *Journal of Hepato-Biliary-Pancreatic Surgery.* 2007;14(6):595-599.
16. Chaugle H, Parchment C, Grotte GJ, Keenan DJM. Hypoglycaemia associated with a solitary fibrous tumour of the pleura. *European Journal of Cardio-thoracic Surgery.* 1999;15(1):84-86.

17. Chode S, Albert SG, Shoemaker JD, Green AL. Estimation of glucose utilization in a type 2 diabetes mellitus patient on insulin analogs with tumor hypoglycemia induced by IGF-II. *Growth Horm IGF Res.* 2016;26:8-10.
18. Christofilis MA, Remacle-Bonnet M, Atlan-Gepner C, et al. Study of serum big-insulin-like growth factor (IGF)-II and IGF binding proteins in two patients with extrapancreatic tumor hypoglycemia, using a combination of Western blotting methods. *Eur J Endocrinol.* 1998;139(3):317-322.
19. Chung JO, Hong SI, Cho DH, Lee JH, Chung DJ, Chung MY. Hypoglycemia associated with the production of insulin-like growth factor II in a pancreatic islet cell tumor: a case report. *Endocr J.* 2008;55(3):607-612.
20. Ciudin A, Lecube A, Hernández C, Mesa J, Simó R. Non-islet cell induced hypoglycemia by "big-IGF-2" in a patient with retroperitoneal solitary fibrous tumor and a papillary thyroid carcinoma: An unusual association. *Endocrinol Nutr.* 2013;60(8):483-484.
21. Cole FH, Jr., Ellis RA, Goodman RC, Weber BC, Courington DP. Benign fibrous pleural tumor with elevation of insulin-like growth factor and hypoglycemia. *South Med J.* 1990;83(6):690-694.
22. Crowley MT, Lonergan E, O'Callaghan P, et al. IGF-2 mediated hypoglycemia and the paradox of an apparently benign lesion: a case report & review of the literature. *BMC Endocr Disord.* 2022;22(1).
23. Daughaday WH, Emanuele MA, Brooks MH, Barbato AL, Kapadia M, Rotwein P. Synthesis and secretion of insulin-like growth factor II by a leiomyosarcoma with associated hypoglycemia. *N Engl J Med.* 1988;319(22):1434-1440.
24. Daughaday WH, Kapadia M. Significance of abnormal serum binding of insulin-like growth factor II in the development of hypoglycemia in patients with non-islet-cell tumors. *Proc Natl Acad Sci U S A.* 1989;86(17):6778-6782.
25. Davda R, Seddon BM. Mechanisms and Management of Non-islet Cell Tumour Hypoglycaemia in Gastrointestinal Stromal Tumour: Case Report and a Review of Published Studies. *Clinical Oncology.* 2007;19(4):265-268.
26. De Groot JWB, Rikhof B, Van Doorn J, et al. Non-islet cell tumour-induced hypoglycaemia: A review of the literature including two new cases. *Endocrine-Related Cancer.* 2007;14(4):979-993.
27. De Los Santos-Aguilar RG, Chavez-Villa M, Contreras AG, et al. Successful multimodal treatment of an IGF2-producing solitary fibrous tumor with acromegaloid changes and hypoglycemia. *Journal of the Endocrine Society.* 2019;3(3):537-543.
28. Deguchi Y, Komuta W, Watanabe T, et al. Successful Surgical Treatment of a Recurrent Pelvic Solitary Fibrous Tumor of Uterine Origin Accompanied by Doege-Potter Syndrome: A Case Report. *American Journal of Case Reports.* 2022;23 (no pagination).
29. Dimitriadis GK, Gopalakrishnan K, Rao R, et al. Severe paraneoplastic hypoglycemia secondary to a gastrointestinal stromal tumour masquerading as a stroke. *Endocrinology, Diabetes and Metabolism Case Reports.* 2015(pagination).
30. Dutta P, Aggarwal A, Gogate Y, et al. Non-islet cell tumor-induced hypoglycemia: A report of five cases and brief review of the literature. *Endocrinology, Diabetes and Metabolism Case Reports.* 2013.
31. Eastman RC, Carson RE, Orloff DG, et al. Glucose utilization in a patient with hepatoma and hypoglycemia. Assessment by a positron emission tomography. *Journal of Clinical Investigation.* 1992;89(6):1958-1963.
32. Eguchi T, Tokuyama A, Tanaka Y, et al. Hypoglycemia associated with the production of insulin-like growth factor II in adrenocortical carcinoma. *Intern Med.* 2001;40(8):759-763.
33. Escobar GA, Robinson WA, Nydam TL, et al. Severe paraneoplastic hypoglycemia in a patient with a gastrointestinal stromal tumor with an exon 9 mutation: A case report. *BMC Cancer.* 2007;7 (no pagination).

34. Fama F, Bouc YL, Barrande G, et al. Solitary fibrous tumour of the liver with IGF-II-related hypoglycaemia. A case report. *Langenbeck's Archives of Surgery.* 2008;393(4):611-616.
35. Filosso PL, Oliaro A, Rena O, Papalia E, Ruffini E, Mancuso M. Severe hypoglycaemia associated with a giant solitary fibrous tumor of the pleura. *Journal of Cardiovascular Surgery.* 2002;43(4):559-561.
36. Föger B, Zapf J, Lechleitner M, Konwalinka G, Patsch JR. Prevention with glucocorticoids of extrapancreatic tumour-hypoglycaemia as a result of increased 'big' insulin-like growth factor II. *Journal of Internal Medicine.* 1994;236(6):692-693.
37. Frystyk J, Skjærbæk C, Zapf J, Ørskov H. Increased levels of circulating free insulin-like growth factors in patients with non-islet cell tumour hypoglycaemia. *DIABETOLOGIA.* 1998;41(5):589-594.
38. Fukasawa Y, Takada A, Tateno M, et al. Solitary fibrous tumor of the pleura causing recurrent hypoglycemia by secretion of insulin-like growth factor II. *Pathol Int.* 1998;48(1):47-52.
39. Fukuda I, Hizuka N, Takano K, Asakawa-yasumoto K, Demura H, Shizume K. Characterization of Insulin-Like Growth Factor II(IGF-II) and IGF Binding Proteins in Patients with Non-Islet-Cell Tumor Hypoglycemia. *Endocrine Journal.* 1993;40(1):111-119.
40. Garla V, Sonani H, Palabindala V, Gomez-Sanchez C, Subauste J, Lien LF. Non-islet Cell Hypoglycemia: Case Series and Review of the Literature. *Front Endocrinol (Lausanne).* 2019;10:316.
41. Gherbon A, Frandes M, Nicula M, Avram A, Timar R. IGF-2 INDUCED HYPOGLYCEMIA ASSOCIATED WITH LUNG SARCOMA. *Acta Endocrinol (Buchar).* 2022;18(2):232-237.
42. Grondin J, Haynes D. Not gist another case of hypoglycemia. *Chest.* 2017;152(4 Supplement 1):A243.
43. Grunenberger F, Bachellier P, Chenard MP, et al. Hepatic and pulmonary metastases from a meningeal hemangiopericytoma and severe hypoglycemia due to abnormal secretion of insulin-like growth factor: a case report. *Cancer.* 1999;85(10):2245-2248.
44. Gullo D, Sciacca L, Parrinello G, Tomaselli L, Vigneri R. Treatment of hemangiopericytoma-induced hypoglycemia with growth hormone and corticosteroids [1]. *Journal of Clinical Endocrinology and Metabolism.* 1999;84(5):1758-1759.
45. Guo W, Ji Y, Guo L, et al. Severe hypoglycemia and finger clubbing in a patient with a BRCA1 mutation in a solitary fibrous tumor: A case report. *Annals of Translational Medicine.* 2021;9(13) (no pagination).
46. Gupta S, Mehta V, Ahuja S, Punj M, Carazas O, Kazzi M. A rare case of hypoglycemia induced by a classic gastrointestinal stromal tumor. *Journal of Community and Supportive Oncology.* 2017;15(3):e176-e177.
47. Hamberg P, de Jong FA, Boonstra JG, van Doorn J, Verweij J, Sleijfer S. Non-islet-cell tumor induced hypoglycemia in patients with advanced gastrointestinal stromal tumor possibly worsened by imatinib. *Journal of clinical oncology : official journal of the American Society of Clinical Oncology.* 2006;24(18):e30-31.
48. Hanif M, Jaiswal V, Naz S, Patel N, Pokhrel NB, Vadiyala MR. Malignant fibroma presenting as a hypoglycemia and coma in a 45-year-old male patient: A case report. *Clinical Case Reports.* 2022;10(11):e6627.
49. Hata T, Tsuruta Y, Takamori S, Shishikura Y. Non-islet cell tumor hypoglycemia at the second recurrence of malignant solitary fibrous tumor in the retroperitoneum and pelvis: A case report. *Case Reports in Oncology.* 2012;5(2):420-427.
50. Hawasli JA, Hopping JR, Hsueh EC. Hypoglycemia with a large retroperitoneal mass - Case report. *International Journal of Surgery Case Reports.* 2014;5(12):1225-1228.

51. Herrmann BL, Saller B, Kiess W, et al. Primary malignant fibrous histiocytoma of the lung: IGF-II producing tumor induces fasting hypoglycemia. *Exp Clin Endocrinol Diabetes.* 2000;108(8):515-518.
52. Hikichi M, Kiriyama Y, Hayashi T, et al. A Hypoglycemia-inducing Giant Borderline Phyllodes Tumor Secreting High-molecular-weight Insulin-Like Growth Factor II: Immunohistochemistry and a Western Blot Analysis. *Intern Med.* 2018;57(2):237-241.
53. Hill D, Livingstone K, Thomson JE, Perry C, Wark G. IGF-II secreting solitary fibrous tumour of the liver presenting with hypoglycaemia. *Scottish Medical Journal.* 2008;53(1).
54. Hino N, Nakagawa Y, Ikushima Y, Yoshida M, Tsuyuguchi M. A case of a giant phyllodes tumor of the breast with hypoglycemia caused by high-molecular-weight insulin-like growth factor II. *Breast Cancer.* 2010;17(2):142-145.
55. Hirai A, Nakanishi R. Solitary fibrous tumor of the pleura with hypoglycemia associated with serum insulin-like growth factor II. *J Thorac Cardiovasc Surg.* 2006;132(3):713-714.
56. Hirai H, Ogata E, Ohki S, et al. Hypoglycemia associated with a gastrointestinal stromal tumor producing high-molecular-weight insulin growth factor ii: A case report and literature review. *Internal Medicine.* 2016;55(10):1309-1314.
57. Hizuka N, Fukuda I, Takano K, Asakawa-Yasumoto K, Okubo Y, Demura H. Serum high molecular weight form of insulin-like growth factor II from patients with non-islet cell tumor hypoglycemia is O-glycosylated. *J Clin Endocrinol Metab.* 1998;83(8):2875-2877.
58. Hodzic D, Delacroix L, Willemsen P, et al. Characterization of the IGF system and analysis of the possible molecular mechanisms leading to IGF-II overexpression in a mesothelioma. *Hormone and Metabolic Research.* 1997;29(11):549-555.
59. Hoekman K, Van Doorn J, Gloudemans T, Maassen JA, Schuller AGP, Pinedo HM. Hypoglycaemia associated with the production of insulin-like growth factor II and insulin-like growth factor binding protein 6 by a haemangiopericytoma. *Clinical Endocrinology.* 1999;51(2):247-253.
60. Hoff AO, Vassilopoulou-Sellin R. The role of glucagon administration in the diagnosis and treatment of patients with tumor hypoglycemia. *Cancer.* 1998;82(8):1585-1592.
61. Holt RIG, Teale JD, Jones JS, Quin JD, McGregor AM, Miell JP. Gene expression and serum levels of insulin-like growth factors (IGFs and IGF-binding proteins in a case of non-islet cell tumour hypoglycaemia. *Growth Horm IGF Res.* 1998;8(6):447-454.
62. Honma H, Takahashi Y, Matsui M, et al. Non-Islet Cell Tumor Hypoglycemia Is Caused by Big IGF-II in a Patient with a Carcinosarcoma of the Uterus. *Intern Med.* 2015;54(24):3165-3169.
63. Höög A, Sandberg Nordqvist AC, Hulting AL, Falkmer UG. High-molecular weight IGF-2 expression in a haemangiopericytoma associated with hypoglycaemia. *APMIS.* 1997;105(6):469-482.
64. Horiuchi T, Shinohara Y, Sakamoto Y, et al. Expression of insulin-like growth factor II by a gastric carcinoma associated with hypoglycaemia. *Vichows Archiv A Pathol Anat.* 1994;424(4):449-452.
65. Hosaka S, Katagiri H, Wasa J, Murata H, Takahashi M. Solitary fibrous tumor in the pelvis: induced hypoglycemia associated with insulin-like growth factor II. *J Orthop Sci.* 2015;20(2):439-443.
66. Hu Y, Mahar TJ, Hicks DG, et al. Malignant solitary fibrous tumor: Report of 3 cases with unusual features. *Applied Immunohistochemistry and Molecular Morphology.* 2009;17(5):451-457.
67. Igarashi Y, Hirukawa H, Nakano T, Morimoto Y, Fukuda S, Tada T. Well-differentiated liposarcoma causing non-islet cell tumor hypoglycemia. *International Cancer Conference Journal.* 2022;11(3):210-214.
68. Ikeda K, Mizuguchi M, Yoshida H, et al. Preclinical Cushing's syndrome associated with non-islet cell tumor hypoglycemia; an additional report. *Internal Medicine.* 2003;42(11):1151-1152.
69. Ishida S, Noda M, Kuzuya N, et al. Big Insulin-Like Growth Factor II-Producing Hepatocellular Carcinoma Associated with Hypoglycemia. *Internal Medicine.* 1995;34(12):1201-1206.

70. Ishihara H, Omae K, Iizuka J, et al. Late recurrence of a malignant hypoglycemia-inducing pelvic solitary fibrous tumor secreting high-molecular-weight insulin-like growth factor-II: A case report with protein analysis. *Oncol Lett.* 2016;12(1):479-484.
71. Izutsu T, Ito H, Fukuda I, et al. Early Improvement of Non-islet Cell Tumor Hypoglycemia by Chemotherapy Using Lenvatinib in a Case with Type 2 Diabetes and Hepatocellular Carcinoma Producing Big IGF-II. *Intern Med.* 2021;60(9):1427-1432.
72. Jannin A, Espiard S, Benomar K, et al. Non-islet-cell tumour hypoglycaemia (NICTH): About a series of 6 cases. *Annales d'Endocrinologie.* 2019;80(1):21-25.
73. Jin K, Zhong S, Lin L, et al. Targeting-intratumoral-lactic-acidosis transcatheter-arterial-chemoembolization for non-islet cell tumor hypoglycemia secondary to a liver metastatic solitary fibrous tumor: A case report and literature review. *Frontiers in Endocrinology.* 2022;13 (no pagination).
74. Kageyama K, Moriyama T, Hizuka N, et al. Hypoglycemia associated with big insulin-like growth factor II produced during development of malignant fibrous histiocytoma. *Endocr J.* 2003;50(6):753-758.
75. Kameyama K, Okumura N, Kokado Y, Miyoshi K, Matsuoka T, Nakagawa T. Solitary Fibrous Tumor Associated With Non-Islet Cell Tumor Hypoglycemia. *Annals of Thoracic Surgery.* 2007;84(1):292-294.
76. Kanzaki M, Kashihara H, Kiura K, et al. Severe hypoglycemia induced by IGF-II producing non-islet cell tumor. *Intern Med.* 2007;46(13):1061.
77. Karki A, Yang J, Chauhan S, Thurm C, Morante J. Paraneoplastic hypoglycemia and paraneoplastic cerebellar degeneration with a solitary fibrous tumor-a case report. *American Journal of Respiratory and Critical Care Medicine Conference: American Thoracic Society International Conference, ATS.* 2017;195(no pagination).
78. Kato A, Bando E, Shinozaki S, et al. Severe hypoglycemia and hypokalemia in association with liver metastases of gastric cancer. *Internal Medicine.* 2004;43(9):824-828.
79. Khaleeli A, Perumainar M, Spedding AV, Teale JD, Marks V. Treatment of tumour-induced hypoglycaemia with human growth hormone. *Journal of the Royal Society of Medicine.* 1992;85(5):303.
80. Khowaja A, Johnson-Rabbett B, Bantle J, Moheet A. Hypoglycemia mediated by paraneoplastic production of Insulin like growth factor-2 from a malignant renal solitary fibrous tumor - clinical case and literature review. *BMC Endocr Disord.* 2014;14:49.
81. Kim SW, Lee SE, Oh YL, Kim S, Park SH, Kim JH. Nonislet Cell Tumor Hypoglycemia in a Patient with Adrenal Cortical Carcinoma. *Case Reports in Endocrinology.* 2016;2016 (no pagination).
82. Kimura S, Mitsuzuka K, Yamada S, et al. Hypoglycemia Caused by Recurrent Renal Cell Carcinoma As Result of Production of High Molecular Weight Insulin-Like Growth Factor 2. *J Clin Oncol.* 2016;34(13):e120-122.
83. Kishi K, Homma S, Tanimura S, Matsushita H, Nakata K. Hypoglycemia induced by secretion of high molecular weight insulin-like growth factor-II from a malignant solitary fibrous tumor of the pleura. *Intern Med.* 2001;40(4):341-344.
84. Kojima G, Terada K, Miki N, Miki K. Non-islet cell tumor hypoglycemia associated with recurrent carcinosarcoma of the ovary. *Endocrine Practice.* 2013;19(4):e83-e87.
85. Kondo S, Hashimoto H, Nakajima K, et al. Insulin-like growth factor II-producing colonic carcinoma presenting with non-islet cell tumor hypoglycemia: An autopsy report revealing neuroendocrine differentiation in the metastatic foci and literature review. *Pathol Int.* 2022;72(3):193-199.
86. Korevaar TIM, Ragazzoni F, Weaver A, Karavitaki N, Grossman AB. Igf2-induced hypoglycemia unresponsive to everolimus. *Qjm.* 2014;107(4):297-300.

87. Krishnan L, Clark J. Non-islet cell tumour hypoglycaemia. *BMJ Case Reports.* 2011.
88. Kuenen BC, Van Doorn J, Slee PHTJ. Non-islet-cell tumour induced hypoglycaemia: A case report and review of literature. *Netherlands Journal of Medicine.* 1996;48(5):175-179.
89. Lawson EA, Zhang X, Crocker JT, Wang WL, Klibanski A. Hypoglycemia from IGF2 overexpression associated with activation of fetal promoters and loss of imprinting in a metastatic hemangiopericytoma. *Molecular Endocrinology.* 2009;23(6):944.
90. Lee AS, Twigg SM. A mitotic cause of Whipple's triad: Non-islet cell tumour hypoglycaemia in incurable low-grade malignancy. *BMJ Case Reports.* 2015;2015 (no pagination).
91. Liu Y, Zhang M, Yang X, et al. Severe Hypoglycemia Caused by a Giant Borderline Phyllodes Tumor of the Breast: A Case Report and Literature Review. *Front Endocrinol (Lausanne).* 2022;13:871998.
92. Loo ML, Harkness R, Watson J, et al. A rare case of insulin-like growth factor (IGF)-2 mediated hypoglycaemia secondary to solitary fibrous tumours. *Diabetic Medicine.* 2018;35(Supplement 1):106.
93. Ma RCW, Tong PCY, Chan JCN, Cockram CS, Chan MHM. A 67-year-old woman with recurrent hypoglycemia: Non-islet cell tumour hypoglycemia. *Cmaj.* 2005;Canadian Medical Association Journal. 173(4):359-361.
94. Marchetti KR, Albergaria Pereira MA, Lichtenstein A, Paiva EF. Refractory hypoglycemia in a patient with functional adrenal cortical carcinoma. *Endocrinology, Diabetes and Metabolism Case Reports.* 2016;2016 (no pagination).
95. Martínez García M, Trincado Aznar P, López Alaminos ME, González Fernández M, Alvarado Rosas A, Laclaustra Gimeno M. Persistent hypoglycemia due to an IGF-II-secreting malignant pheochromocytoma: a case report and literature review. *Clin Case Rep.* 2020;8(12):2433-2435.
96. Maruyama H, Tatsumi M, Kitayama H, et al. A case of gastric cancer with non-islet cell tumor hypoglycemia detected by insulin-like growth factor II. *Pathol Int.* 2010;60(8):595-597.
97. Masson EA, MacFarlane IA, Graham D, Foy P. Spontaneous hypoglycaemia due to a pleural fibroma: Role of insulin like growth factors. *Thorax.* 1991;46(12):930-931.
98. Mathez ALG, Moroto D, Dib SA, De Sa JR. Seborrheic keratoses and severe hypoinsulinemic hypoglycemia associated with insulin grow factor 2 secretion by a malignant solitary fibrous tumor. *Diabetology and Metabolic Syndrome.* 2016;8(1) (no pagination).
99. Matsuda S, Usui M, Sakurai H, Suzuki H, Ogura Y, Shiraishi T. Insulin-like growth factor II-producing intra-abdominal hemangiopericytoma associated with hypoglycemia. *J Gastroenterol.* 2001;36(12):851-855.
100. Mehta SK, Singh R, Prasad SK, Pandey N. A rare case of IGF2 mediated hypoglycemia in a diabetic patient - A praneoplastic manifestation of an adrenal tumor. *Polish Annals of Medicine.* 2018;25(2):237-240.
101. Mon A, Ahluwalia R, Tehseen S, Rathore A, Weston P, Ahmad A. A rare case of steroid responsive non-islet cell tumour hypoglycemia (NICTH) secondary to metastatic hemangiopericytoma. *Endocrine Abstracts.* 2010;21:P202.
102. Morbois-Trabut L, Maillot F, De Widerspach-Thor A, Lamisse F, Couet C. "Big IGF-II"-induced hypoglycemia secondary to gastric adenocarcinoma. *Diabetes Metab.* 2004;30(3):276-279.
103. Mukherjee S, Diver M, Weston PJ. Non islet cell tumor hypoglycaemia in a metastatic Leydig cell tumor. *Acta Oncologica.* 2005;44(7):761-763.
104. Nagasaka Y, Lee AA, Rho J, Cox KA. An 85 year old woman presenting with whipple's triad for hypoglycemia. *Journal of General Internal Medicine.* 2012;2):S387.
105. Nalbani M, Simetic L. Doege-Poter syndrome: Hypoglycemia secondary to solitary fibrous tumors; Case report. *Libri Oncologici.* 2018;45(Supplement 2):35.

106. Nanayakkara PWB, Van Doorn J, Van den Berg FG, Van Groeningen CJ, Pinedo HM, Hoekman K. Treatment of haemangiopericytoma-associated hypoglycaemia with embolisation. *European Journal of Internal Medicine.* 2002;13(5):340-343.
107. Nicolaou V, Shires R, Huddle KRL. Double jeopardy: Hypoglycaemia and advanced hepatocellular carcinoma. *Journal of Endocrinology, Metabolism and Diabetes of South Africa.* 2013;18(2):120-122.
108. North AS, Thakkar RG, James RA, Hammond JS. The palliative management of non-islet cell tumour hypoglycaemia with glucocorticoids and somatostatin analogues in an unresectable hepatocellular carcinoma. *Annals of the Royal College of Surgeons of England.* 2022;104(6):e180-e182.
109. Ogunsakin AA, Hilsenbeck HL, Portnoy DC, Nyenwe EA. Recurrent Severe Hypoinsulinemic Hypoglycemia Responsive to Temozolomide and Bevacizumab in a Patient With Doege-Potter Syndrome. *American Journal of the Medical Sciences.* 2018;356(2):181-184.
110. Ono M, Maeda Y, Koyama N, et al. A case of nonislet cell tumor hypoglycemia associated with malignant mesothelioma requiring a multifaceted approach for optimal glycemic control. *Clinical Case Reports.* 2021;9(10) (no pagination).
111. Otake S, Kikkawa T, Takizawa M, et al. Hypoglycemia Observed on Continuous Glucose Monitoring Associated With IGF-2-Producing Solitary Fibrous Tumor. *J Clin Endocrinol Metab.* 2015;100(7):2519-2524.
112. Pacioles T, Seth R, Orellana C, Dhaliwal R. Malignant phyllodes tumor of the breast presenting with hypoglycemia: A case report and literature review. *Cancer Management and Research.* 2014;6:467-473.
113. Perinkulam Sathyanarayanan S, Anel-Tiangco RML, Tiangco NDL. Doege-Potter syndrome in a patient with solitary fibrous tumor of the lung: A rare cause of recurrent hypoglycemia. *Journal of Clinical and Translational Endocrinology: Case Reports.* 2022;24 (no pagination).
114. Pink D, Schoeler D, Lindner T, et al. Severe hypoglycemia caused by paraneoplastic production of IGF-II in patients with advanced gastrointestinal stromal tumors: a report of two cases. *J Clin Oncol.* 2005;23(27):6809-6811.
115. Plikat K, Reichle A, Elmlinger MW, Scholmerich J. Hypoglycemia associated with the production of insulin-like growth factor (IGF)-II by a hemangiopericytoma. [German]. *Deutsche Medizinische Wochenschrift.* 2003;128(6):257-260.
116. Ramos N, Ramos R, Marialva C, Silva E. An urological cause of hypoglycaemia: A case report of the Doege-Potter syndrome. *Archivio Italiano di Urologia e Andrologia.* 2020;92(4):392-393.
117. Rana P, Kim B. A Unique Case of IGF-2 Induced Hypoglycemia Associated with Hepatocellular Carcinoma. *Case Rep Endocrinol.* 2019;2019:4601484.
118. Ratoff JC, Gossage AA. Extrapancreatic tumour hypoglycaemia. *Hospital medicine (London, England : 1998).* 2000;61(12):866-867.
119. Renard E, Langbour-Remy C, Klein M, Le Bouc Y, Weryha G, Cuny T. Severe hypoglycemia with "Big"-IGF-2 oversecretion by a giant phyllode tumor of the breast: a rare case of non-islet cell tumor-induced hypoglycemia (NICTH). *Ann Endocrinol (Paris).* 2012;73(5):488-491.
120. Richters L, Ortmann M, Faust M, et al. The oncological emergency case: Paraneoplastic hypoglycemia in metastatic breast cancer - Case report and brief review of the literature. *Breast Care.* 2013;8(5):368-370.
121. Rikhof B, Van Den Berg G, Van Der Graaf WTA. Non-islet cell tumour hypoglycaemia in a patient with a gastrointestinal stromal tumour [1]. *Acta Oncologica.* 2005;44(7):764-766.
122. Ron D, Powers AC, Pandian MR, Godine JE, Axelrod L. Increased insulin-like growth factor II production and consequent suppression of growth hormone secretion: a dual mechanism for tumor-induced hypoglycemia. *J Clin Endocrinol Metab.* 1989;68(4):701-706.

123. Rosario PW, Furtado MS, Castro AF, Purisch S. Non-islet cell tumor hypoglycemic in a patient with poorly differentiated thyroid cancer [2]. *Thyroid.* 2007;17(1):84-85.
124. Rosseel L, De Leu N, Van Hecke W, Unuane D. A rare case of hypoglycemia in a patient with elevated right hemidiaphragm. *BMJ Case Reports.* 2012.
125. Ruiz García I, Sánchez Torralvo FJ, Contreras Bolívar V. IGF-2-mediated resistant hypoglycemia in a patient with a nonislet cell tumor. *Med Clin (Barc).* 2021;157(4):208-209.
126. Saeed Z, Taleb S, Evans-Molina C. A case of extragastrointestinal stromal tumor complicated by severe hypoglycemia: A unique presentation of a rare tumor. *BMC Cancer.* 2016;16(1) (no pagination).
127. Saito Y, Suzuki Y, Inomoto C, et al. A case of giant borderline phyllodes tumor of the breast associated with hypoglycemia. *Tokai Journal of Experimental and Clinical Medicine.* 2016;41(3):118-122.
128. Sakamoto T, Kaneshige H, Takeshi A, Tsushima T, Hasegawa S. Localized pleural mesothelioma with elevation of high molecular weight insulin-like growth factor II and hypoglycemia. *Chest.* 1994;106(3):965-967.
129. Samlani-Sebbane Z, Diffaa A, Krati K, et al. Fatal hypoglycaemia from IGF II hyperproduction as a complication of a mesenteric gastrointestinal stromal tumour. *Arab Journal of Gastroenterology.* 2011;12(3):171-172.
130. Sato R, Tsujino M, Nishida K, et al. High molecular weight form insulin-like growth factor II-producing mesenteric sarcoma causing hypoglycemia. *Intern Med.* 2004;43(10):967-971.
131. Schofield PN, Connor H, Turner RC, Zapf J. Tumour hypoglycaemia: Raised tumour IGFII mRNA associated with reduced plasma somatomedins. *British Journal of Cancer.* 1989;60(5):661-663.
132. Schweichler M, Hennessey JV, Cole P, Perdue JF, Le Roith D. Hypoglycemia in pregnancy secondary to a non-islet cell tumor of the pleura and ectopic insulin-like growth factor II hormone production. *Obstet Gynecol.* 1995;85(5 Pt 2):810-813.
133. Sharma M, Chang PEJ, Tan CK, Sekar R, Lee PC. Refractory hypoglycemia presenting as a paraneoplastic manifestation of advanced hepatocellular carcinoma. *Proceedings of Singapore Healthcare.* 2012;1):S235.
134. Sierra-Poyatos R, Cardenas-Salas J, Ortega-Juaristi M, Vazquez-Martinez C. About a rare case of hypoglycemia: Non-islet cell tumor hypoglycemia (NICTH). *Endocrinologia, diabetes y nutricion.* 2021;68(8):589-591.
135. Simpson V, McGovern A. Recurrent hypoglycemia and a slowly rising hemidiaphragm: A case report. *Clinical Case Reports.* 2021;9(7) (no pagination).
136. Slee PHTJ, Beckers MMJ, Van Doorn J. Hypoglycaemia in a patient with a gastrointestinal stromal tumour [1]. *Clinical Endocrinology.* 2003;59(3):402-404.
137. Sohda T, Yun K. Insulin-like growth factor II expression in primary meningeal hemangiopericytoma and its metastasis to the liver accompanied by hypoglycemia. *Hum Pathol.* 1996;27(8):858-861.
138. Soran H, Younis N, Joseph F, Hayat Z, Zakhour H, Scott A. A case of haemangiopericytoma-associated hypoglycaemia: Beneficial effect of treatment with radiotherapy. *International Journal of Clinical Practice.* 2006;60(10):1319-1322.
139. Stein RH, Herman SD, Phelps RG, Sapadin AN. Florid eruption of seborrheic keratoses associated with elevated insulin-like growth factor, hypoglycemia, and solitary fibrous tumor of the pleura. *Int J Dermatol.* 2004;43(12):944-947.
140. Strauss G, Christensen L, Zapf J. Tumour-induced hypoglycaemia due to 'big' IGF-II. *Journal of Internal Medicine.* 1994;236(1):97-99.

141. Sturrock NDC, Selby C, Hosking DJ. Spontaneous hypoglycaemia in a non-insulin-dependent diabetes mellitus patient with disseminated pancreatic carcinoma. *Diabetic Medicine.* 1997;14(4):324-326.
142. Takebayashi K, Furukawa S, Okumura T, et al. Severe Non-Islet Cell Hypoglycemia From Ileum-Origin Gastrointestinal Stromal Tumor Producing Insulin-Like Growth Factor-2 in a Patient With Liver Cirrhosis Due to Chronic Hepatitis B. *J Clin Med Res.* 2020;12(12):824-830.
143. Tay CKJ, Teoh HL, Su S. A common problem in the elderly with an uncommon cause: Hypoglycaemia secondary to the Doege-Potter syndrome. *BMJ Case Reports.* 2015;2015 (no pagination).
144. Teale JD, Blum WF, Marks V. Alleviation of non-islet cell tumour hypoglycaemia by growth hormone therapy is associated with changes in IGF binding protein-3. *Annals of Clinical Biochemistry.* 1992;29(3):314-323.
145. Teale JD, Marks V. INAPPROPRIATELY ELEVATED PLASMA INSULIN-LIKE GROWTH FACTOR II IN RELATION TO SUPPRESSED INSULIN-LIKE GROWTH FACTOR I IN THE DIAGNOSIS OF NON-ISLET CELL TUMOUR HYPOGLYCAEMIA. *Clinical Endocrinology.* 1990;33(1):87-98.
146. Teale JD, Marks V. Glucocorticoid therapy suppresses abnormal secretion of big IGF-II by non-islet cell tumours inducing hypoglycaemia (NICTH). *Clinical Endocrinology.* 1998;49(4):491-498.
147. Teale JD, Wark G. The effectiveness of different treatment options for non-islet cell tumour hypoglycaemia. *Clinical Endocrinology.* 2004;60(4):457-460.
148. Tee HC, Valayatham VM. Recurrent severe hypoglycemia secondary to benign phyllodes tumor of the breast: A rare case of non-islet cell tumor-induced hypoglycemia (NICTH). *Journal of the ASEAN Federation of Endocrine Societies.* 2021;36(2).
149. Teramae S, Miyamoto H, Muguruma N, et al. Insulin-like growth factor II-producing metastatic colon cancer with recurrent hypoglycemia. *Clin J Gastroenterol.* 2015;8(1):35-40.
150. Thomas J, Kumar SC. Nonislet cell tumor hypoglycemia. *Case Reports in Endocrinology.* 2013;2013 (no pagination).
151. Tietge UJF, Schofl C, Ocran KW, et al. Hepatoma with severe non-islet cell tumor hypoglycemia. *American Journal of Gastroenterology.* 1998;93(6):997-1000.
152. Tiruchelvam N, Kistangari G, Listinsky C, Daw H, Kumar V. Life-threatening hypoglycemia resulting from a non-islet cell tumor. *Journal of Community and Supportive Oncology.* 2015;13(8):296-297.
153. Tominaga N, Kawarasaki C, Kanemoto K, et al. Recurrent solitary fibrous tumor of the pleura with malignant transformation and non-islet cell tumor-induced hypoglycemia due to paraneoplastic overexpression and secretion of high-molecular-weight insulin-like growth factor II. *Intern Med.* 2012;51(23):3267-3272.
154. Tong J, Athayde J, MacKenzie S, Ho M. Successful multidisciplinary treatment of Doege-Potter syndrome: Hypoglycaemia caused by paraneoplastic IGF-2 production by a metastatic haemangiopericytoma. *BMJ Case Reports.* 2021;14(4).
155. Tsirlin A, Borensztein A, Sacerdote AS, Bahtiyar G. IGF-2 related non-islet cell tumor hypoglycemia in hepatocellular carcinoma treated with diazoxide and hydrocortisone. *Endocrine Reviews Conference: 95th Annual Meeting and Expo of the Endocrine Society, ENDO.* 2013;34(3 SUPPL. 1).
156. Tsuro K, Kojima H, Okamoto S, et al. Glucocorticoid therapy ameliorated hypoglycemia in insulin-like growth factor-II-producing solitary fibrous tumor. *Intern Med.* 2006;45(8):525-529.
157. van den Berg SAA, Krol CG. Pro-IGF2-induced hypoglycaemia associated with hepatocellular carcinoma. *Endocrinology, Diabetes and Metabolism Case Reports.* 2017;2017 (no pagination).
158. van Doorn J, van de Hoef W, Dullaart RPF. Quantitative analysis of the concentrations of IGFs and several IGF-binding proteins in a large fibrous abdominal tumor and the circulation of a patient with hypoglycemia. *BioFactors.* 2015;41(3):183-189.

159. Versluis J, Valk G, van Rossum H, Tesselaar M. Non-islet cell tumour hypoglycaemia in a patient with a well-differentiated gastric neuroendocrine tumour. *BMJ Case Reports.* 2019;12(9) (no pagination).
160. Vidyarthi M, Hossain B, Marouf E, Khatami Z, Stojanovic ND. The clinical course of a non-islet cell tumor causing hypoglycemia: A complex case of doege-potter syndrome. *Endocrine Reviews Conference: 93rd Annual Meeting and Expo of the Endocrine Society, ENDO.* 2011;32(3 Meeting Abstracts).
161. Wagner S, Greco F, Hamza A, Hoda RM, Holzhausen HJ, Fornara P. Retroperitoneal malignant solitary fibrous tumor of the small pelvis causing recurrent hypoglycemia by secretion of insulin-like growth factor 2. *Eur Urol.* 2009;55(3):739-742.
162. Wakami K, Tateyama H, Kawashima H, et al. Solitary fibrous tumor of the uterus producing high-molecular-weight insulin-like growth factor II and associated with hypoglycemia. *Int J Gynecol Pathol.* 2005;24(1):79-84.
163. Wasada T, Hizuka N, Yamamoto M, et al. An insulin-like growth factor II-producing histiocytoma associated with hypoglycemia: analysis of the peptide, its gene expression, and glucose transporter isoforms. *Metabolism.* 1992;41(3):310-316.
164. Wong WK. Non-islet cell tumour hypoglycaemia (NICTH) in Malignant Mesothelioma: Case report. *Malaysian Journal of Medical Sciences.* 2015;22(4):81-85.
165. Yamaguchi M, Kamimura S, Takada J, et al. Case report: Insulin-like growth factor II expression in hepatocellular carcinoma with alcoholic liver fibrosis accompanied by hypoglycaemia. *J Gastroenterol Hepatol.* 1998;13(1):47-51.
166. Yamakawa-Yokota F, Ozaki N, Okajima A, Nishio H, Nagasaka T, Oiso Y. Retroperitoneal solitary fibrous tumor-induced hypoglycemia associated with high molecular weight insulin-like growth factor II. *Clin Med Res.* 2010;8(3-4):159-162.
167. Yamasaki H, Itawaki A, Morita M, et al. A case of insulin-like growth factor 2-producing gastrointestinal stromal tumor with severe hypoglycemia. *BMC Endocr Disord.* 2020;20(1):60.
168. Yang CY, Chou CW, Hao LJ. Malignant solitary fibrous tumor with hypoglycemia (Doege-Potter syndrome). *Journal of Postgraduate Medicine.* 2013;59(1):64-66.
169. Yonei Y, Tanaka M, Ozawa Y, et al. Primary hepatocellular carcinoma with severe hypoglycemia; involvement of insulin-like growth factors. *Liver.* 1992;12(2):90-93.
170. Yoshida D, Sugisaki Y, Tamaki T, et al. Intracranial malignant meningioma with abdominal metastases associated with hypoglycemic shock: A case report. *Journal of Neuro-Oncology.* 2000;47(1):51-58.
171. Zachariah S, Brackenridge A, Shojaee-Moradie F, Camuncho-Hubner C, Umpleby AM, Russell-Jones D. The mechanism of non-islet cell hypoglycaemia caused by tumour-produced IGF-II [1]. *Clinical Endocrinology.* 2007;67(4):637-638.
172. Zhou Z, Wei W, Tu J, Jiang Q. Non-islet cell tumor hypoglycemia caused by breast tumor: A case report. *Medicine (United States).* 2021;100(48) (no pagination).
